# A Novel Detection and Multi-Classification Approach for IoT-Malware Using Random Forest Voting of Fine-Tuning Convolutional Neural Networks

**DOI:** 10.3390/s22114302

**Published:** 2022-06-06

**Authors:** Safa Ben Atitallah, Maha Driss, Iman Almomani

**Affiliations:** 1 RIADI Laboratory, University of Manouba, Manouba 2010, Tunisia; safa.benatitallah@ensi-uma.tn; 2 Security Engineering Lab, CCIS, Prince Sultan University, Riyadh 12435, Saudi Arabia; imomani@psu.edu.sa; 3 Computer Science Department, King Abdullah II School for Information Technology, The University of Jordan, Amman 11942, Jordan

**Keywords:** IoT-malware, detection, multi-classification, transfer learning, ensembling strategies, CNNs, random forest voting

## Abstract

The Internet of Things (IoT) is prone to malware assaults due to its simple installation and autonomous operating qualities. IoT devices have become the most tempting targets of malware due to well-known vulnerabilities such as weak, guessable, or hard-coded passwords, a lack of secure update procedures, and unsecured network connections. Traditional static IoT malware detection and analysis methods have been shown to be unsatisfactory solutions to understanding IoT malware behavior for mitigation and prevention. Deep learning models have made huge strides in the realm of cybersecurity in recent years, thanks to their tremendous data mining, learning, and expression capabilities, thus easing the burden on malware analysts. In this context, a novel detection and multi-classification vision-based approach for IoT-malware is proposed. This approach makes use of the benefits of deep transfer learning methodology and incorporates the fine-tuning method and various ensembling strategies to increase detection and classification performance without having to develop the training models from scratch. It adopts the fusion of 3 CNNs, ResNet18, MobileNetV2, and DenseNet161, by using the random forest voting strategy. Experiments are carried out using a publicly available dataset, MaleVis, to assess and validate the suggested approach. MaleVis contains 14,226 RGB converted images representing 25 malware classes and one benign class. The obtained findings show that our suggested approach outperforms the existing state-of-the-art solutions in terms of detection and classification performance; it achieves a precision of 98.74%, recall of 98.67%, a specificity of 98.79%, F1-score of 98.70%, MCC of 98.65%, an accuracy of 98.68%, and an average processing time per malware classification of 672 ms.

## 1. Introduction

The Internet of Things (IoT) is a new paradigm that has gained a lot of traction in recent years due to the adoption of a variety of cutting-edge technologies and communication methods [1,2]. The fundamental concept of IoT is the ubiquitousness of a variety of things, such as smart devices, sensors, actuators, and Radio-Frequency Identification (RFID) tags, among others, that interact and communicate with one another to attain a specific purpose. The IoT allows things to communicate with each other over the internet, and it is fast gaining prominence due to its significant and positive impact on practically every aspect of users’ lives and behavior. However, the rapid proliferation of IoT has brought several issues, one of which is malware attacks. To satisfy the fast-growing demands for IoT devices, some manufacturers are mass-producing and distributing IoT devices that are vulnerable to security breaches. The expansion of these vulnerable devices makes them a principal target for malware developers. Malware might not only leak user data acquired from IoT ecosystems, but it could also access critical networks, allowing it to spread quickly to other networks and information systems. According to Kaspersky, the cybersecurity product developer, 1.51 billion breaches of IoT devices occurred between January and June 2021, up from 639 million in 2020 [3]. Kaspersky also reported that during 2021 more than 872 million IoT assaults were recorded, with the goal of bitcoin mining, distributed denial-of-service (DDoS) shutdowns, or data breaches [3].

Several research on the detection and classification of IoT-malware have recently been conducted in order to reduce the harm caused by malware assaults whilst also protecting IoT devices against new and variant malware attacks [4,5]. Due to the destructive nature of IoT-malware and the difficulties in reversing a malware infection, in this work, we aim to propose a novel and efficient approach of detecting and classifying a malware attack before it infects an IoT ecosystem. It should be noted that malware changes frequently and newer versions of malware families behave differently from their predecessors. This fact makes it difficult for traditional detection methods to detect them. This is supported by the work of Baig et al. [6], which provides an overview of techniques used by malware developers to avoid traditional static detection methods. Machine Learning (ML) and Deep Learning (DL) techniques have been found to be more efficient than static code analysis techniques, as demonstrated in [7,8]. Signature-based techniques are prone to be tricked, especially with regard to newer malware variants. According to [9], just 6% of the ransomware infections that were carried out (e.g., about 62% of the infections of the Angler exploit kit to deliver ransomware) were discovered in VirusTotal out of the 3000 exploit kits analyzed. DL-based ransomware detection follows a very precise learning workflow. First, the data must be organized by features, which can be performed using custom feature selection methods or predetermined algorithms. After this is completed and the best feature set is selected, the data is fed into the DL algorithm. This algorithm will be trained before being subjected to the testing phase. It will require a training set, in the case of ransomware data samples of both benign and ransomware, so that the algorithm can learn to differentiate between these two classes.

Recently, Transfer Learning (TL) methodology has received a lot of traction in a variety of sectors and applications [10,11,12]. Its goal is to reuse previously trained models as the basis for new tasks. This enhances performance on related issues while also speeding up training [13]. One of the most prominent and well-studied applications of DL and TL is image classification. TL for image classification is based on the idea that if a model is trained on a large and general enough dataset, it may successfully serve as a generic model of the visual world. You may then use the learned feature maps to train a large model on a large dataset without having to start from scratch. To customize a pre-trained model, the fine-tuning method is extensively used. It entails unfreezing a few of the top layers of a frozen model base and simultaneously training the newly added classifier layers and the base model’s final layers at the same time [14]. This allows us to “fine-tune” the base model’s higher-order feature representations to make them more relevant for the task. By incrementally adapting the pre-trained features to the new data, this method has the potential to achieve considerable improvements.

The ensembling strategy is another method for improving the performance of DL models. Ensembling is the process of merging various learning algorithms in order to gain their combined performance, i.e., to increase the performance of current models by combining numerous models into a single effective model [15,16]. Ensemble learning implies combining numerous models in some way, such as averaging or voting, so that the ensemble model outperforms any of the individual models. Combining decisions from many models has been shown to be an effective method for improving model performance.

To take advantage of the significant capabilities offered by the TL methodology in the image classification domain, we propose to use the visualization approach to ensure malware detection and classification. Malware visualization is a technique for converting malicious software into an image by extracting its binaries [17]. Each malicious family has a unique texture pattern in the produced images of malware apps. The malware visualization analysis also has the benefit of requiring no static compilation or dynamic execution of malware programs. After the malware visualization phase, the training classifier is performed using the malware image’s textural features. As a result, even if the attacker used obfuscation or modification tools and techniques, the malware image will display the texture representing the malicious program [18].

In this paper, we propose a vision-based malware multi-classification approach to address the inadequacies of current malware detection systems. The proposed approach makes use of the benefits of deep TL methodology and incorporates the fine-tuning method and various ensembling strategies to increase detection and classification performance without having to develop the training models from scratch. It adopts the fusion of 3 Convolutional Neural Networks (CNNs), ResNet18, MobileNetV2, and DenseNet161, by using the random forest voting strategy. The main contributions in this paper are summarized in the following points:

- Apply TL using the fine-tuning method for different pre-trained CNN models and combine the features extracted by the pre-trained models using different ensemble strategies, namely voting, stacking, and decision fusion strategies, to provide more accurate classification results;
- Validate the proposed approach by using a public dataset, MaleVis, which is made of more than 14,000 RGB images representing 26 distinct families. It includes 25 classes of five different malware types (i.e., Adware, Trojan, Virus, Worm, Backdoor) and one benign class;
- Conduct rigorous performance analysis in terms of distinct performance evaluation metrics to correctly assess the fine-tuned CNN models under consideration;
- Compare the experimental results of the applied ensemble strategies to decide about the most appropriate strategy to be adopted for the considered dataset and the malware detection and multi-classification tasks;
- Conduct a comparative analysis of the suggested approach’s performance with other approaches published in recent relevant works and using the same considered dataset.

The remainder of the paper is organised as follows. Section 2 highlights recent state-of-the-art solutions for malware detection and classification utilizing DL, vision-based techniques, and adopting the TL methodology. Section 3 presents the proposed vision-based malware multi-classification approach. Section 4 covers implementation and experimental analysis. Section 5 provides a summary of this study as well as future research directions.

## 2. Related Work

Many malware detection and classification research works have been conducted using various analytical methodologies [19,20,21]. Extensive research on malware classification has recently been conducted using DL and vision-based techniques. This section provides a detailed overview of recent and relevant malware classification approaches using these two techniques and adopting the TL methodology.

In [22], Lo et al. suggested a method for classifying malware families using a deep CNN based on the Xception architecture. The TL technique was used in this work to deploy the Xception model to ensure malware classification. Furthermore, the suggested method employs a CNN model to automatically extract image features, which is significantly faster than traditional approaches that rely on manual feature engineering. The proposed method consists of three steps: (1) Malware visualization, (2) Xception model training using two different types of files (i.e., .bytes and .asm), and (3) stacking and training the outputs obtained from the two distinct types of files by employing an ensemble model to get superior performance results.

An investigation about efficiency of three CNN-based models (i.e., AlexNet, ResNet, and VGG16) as classification tools and feature extractors following malware visualization is presented in [23]. The authors propose a novel CNN model that can be used as a classifier and as a feature extractor. In this work, Davuluru et al. suggest combining the pattern recognition technique that has proved its efficiency for malware classification with CNN, which has been producing cutting-edge results for image-based classification. They proceeded by extracting features using the proposed CNN architectures and categorizing the obtained results using classic ML methods like Support Vector Machine (SVM) and k-Nearest Neighbors (kNN). Adopting a fusion-based method, which aggregates all the probabilities offered by all CNN-based models, resulted in a performance improvement.

In [24], An upgraded Faster-RCNN (Region-Convolutional Neural Networks) developed by applying TL to the malware detection task using code texture analysis is presented. In this work, the authors used code visualization to illustrate malicious behaviors in order to detect malware programs. To efficiently counteract code obfuscation, the authors use CNN to obtain particular elements of malware texture visual analysis and apply Region Proposal Network (RPN) to locate these texture’s key features. Then, to speed up convergence, the Faster RCNN model was applied as a malware classification model. Simultaneously, the authors proposed a new objective function for resolving image distortion and overfitting after TL. To validate the proposed approach, the authors collected code fragments from six malware families and evaluated the experimental findings before and after applying TL.

Three distinct ways to classifying malware programs based on different file formats are described and investigated in [25]: (a) A CNN-based approach, including AlexNet, ResNet, and VGG-16 architectures, for categorizing malware generated files after rendering them as images, (b) A Recurrent Neural Network (RNN)-based approach for classifying malware assembly files, and (c) An ensemble approach for classifying malware assembly files that combines the features extracted using (a) and (b) techniques and then classifying them using Logistic Regression (LR) or Support Vector Machine (SVM). The main benefit of using the LR or SVM ML models was to integrate both sequential and graphical techniques, which helped in producing a high performance with minimal memory usage.

The work presented in [26] proposed an Image-based Malware Classification approach based on an Ensemble of CNN architectures (IMCEC), which entails adapting and fine-tuning information from several CNN models that have already been trained with ImageNet data to ensure the classification of malware images. The obtained features were used to train several multiclass classifiers that were trained utilizing the transferred features and fused posterior probabilities to increase the classification accuracy of families of unfamiliar malware samples. Even under obfuscation attacks, the proposed IMCEC properly categorized the majority of malware families and outperformed other algorithms using similar benchmarks.

In [27], the authors looked at how CNN and VGG16 models may be used to classify malware into nine separate families. The original malware dataset, which consisted of hexadecimal byte representations of each malware file, was translated into decimal equivalents first. To represent the RGB values of an image pixel, these decimal numbers were then arranged into three classes. Following that, the pixels were converted into image files. The CNN and VGG16 models both used these image files as inputs. Five series of experiments were carried out and scored using a set of performance metrics. When compared to the CNN neural network, the experiments revealed that using DL in the form of the fine-tuned VGG16 model produced higher performance results.

In [28], Awan et al. present a DL system based on spatial attention and convolutional neural networks, named SACNN, ensuring the categorization of 25 well-known malware families with and without class balancing. The suggested model architecture was divided into three blocks. The first block was a VGG19 TL model based on ImageNet. The CNN model, which had been boosted by attention, was the next block of the model architecture. Dynamic spatial convolution is a technique used to create attention. It’s a form of spatial attention that’s particularly useful for image analysis. Because not all portions of an image are equally important, dynamic spatial convolution uses a straightforward global average pooling technique. Fully connected layers made up the last block of the model architecture. The experiments were conducted using a well-known benchmark dataset and showed the effectiveness of the proposed architecture in classifying malware programs. SACNN was also tested on non-malware classes, and it proved to be quite effective in detecting malware from image files.

Kumar presented in [29] a novel convolution neural networks model, named MCFT-CNN, for malware classification. Even when sophisticated evading tactics were used to generate the malware, the MCFT-CNN model recognized it without feature engineering or prior knowledge of binary code examination or reverse engineering. The suggested model used deep TL to categorize malware images into their different malware families. The suggested model improved on the ResNet50 model by replacing the last layer with a fully connected dense layer. The softmax layer received the output of the fully connected dense layer as well as the knowledge of the ImageNet model for malware classification. The suggested model showed consistent efficiency on two benchmark datasets, demonstrating the model’s comprehensiveness to perform on a wide range of datasets.

In [30], The authors addressed a consistency study against obfuscation performed on four CNNs, namely ResNet50, InceptionV3, VGG16, and MobileNet, which are frequently utilized for developing image-based malware classification systems. To that end, the authors proposed to retrain the CNN models using TL to classify malware from 9 distinct families using a well-known dataset benchmark. The experimental results showed that the considered image-based techniques achieved excellent accuracy with a small loss on obfuscated samples. MobileNet, specifically, had demonstrated great accuracy and resilience, as well as a very rapid classification time.

To assure malware detection and classification, the authors in [31] presented a malware classification framework based on a CNN architecture. They proposed also to integrate the SMOTE algorithm (Synthetic Minority Oversampling Technique) to improve the framework’s performance. The suggested solution entailed converting binary data to grayscale images, balancing them using the SMOTE method, and then training the CNN architecture to detect and recognize malware families. The authors employed the TL approach, which is based on the VGG16 DL model. This model was previously trained on a huge dataset benchmark. A thorough experiment was conducted utilizing a well-known Malware dataset for assessments. The findings have shown that the proposed architecture provided good results and could be utilized to fix the CNN models’ effectiveness decrease while dealing with imbalanced malware families.

Work [32] presented a method for converting malware compiled codes into their visual images and obtaining grayscale images of malicious codes using a visual malware classification algorithm. After passing the grayscale images through deep convolutional neural networks, the categorization of malicious codes into their corresponding malware types was obtained. In this work, the authors compared the performance of many benchmarked “ImageNet” models, including VGG16, VGG19, Xception, InceptionV3, DenseNet201, InceptionResNetV2, ResNet50, NASNetLarge, MobileNetV2 and AlexNet. TL methodology was adopted by using these pre-trained models. For the experimentation, the authors utilized a malware dataset with 25 samples that have been benchmarked. The proposed technique for image-based malware classification was shown to be effective throughout the experiments.

To categorize distinct imbalanced families of malware images, the work presented in [33] introduced a DL-based visualized malware multiclassification architecture. This architecture was created using well-developed malware imaging, fine-tuning, and CNN-based TL techniques to accurately identify various malware families. VGG16, AlexNet, DarkNet-53, DenseNet-201, Inception-V3, Places365-GoogleNet, ResNet-50, and MobileNet-V2 are eight fine-tuned CNN models that were previously tested on the ImageNet database and used in this work. The proposed approach’s key contribution is its cost-effectiveness in dealing with unbalanced malware types while attaining good detection accuracy without the requirement for data augmentation or sophisticated feature engineering. By using a well-known unbalanced benchmark dataset, extensive experiments based on several performance measures were carried out, demonstrating the proposed architecture’s remarkable classification capabilities and competency.

In [34], a suggested approach called DTMIC, which stood for Deep TL for Malware Image Classification, was used to classify malware using the capabilities offered by the deep CNN architecture previously trained using the ImageNet dataset. In this work, the Portable Executable files (PEs) in Windows were transformed to grayscale images, based on the assumption that comparable malware families have similar features when they are displayed. Grayscale images are then passed to an improved CNN model. The retrieved characteristics were flattened and placed in a dense layer that was entirely connected. To prevent the overfitting problem that many CNN models have, a normalization technique called Early Stopping was employed to monitor the validation loss with appropriate constraints. Using two benchmark datasets, the model’s efficacy and robustness were assessed. The experimental results have shown that the suggested DTMIC approach outperformed the baseline models and was robust to both packed and encrypted malware.

In summary, DL approaches for detecting and classifying malware intrusions from features transformed to images are becoming much more popular, and a wide range of neural network models and architectures are being explored, improved, and implemented. Nonetheless, with so many different DL architectures and hyperparameters to choose from, more investigation is necessary to uncover the optimal solutions for the cybersecurity area. We may conclude the following weaknesses from our investigation of the previously presented relevant works:

- Because of the similarity of features in some malware families, the presented results of some related works that used the same datasets to assess their suggested approaches suffer from a high rate of misclassification of particular malware families and classes [25,28].
- Previously presented studies are time-consuming and provide a high level of complexity to assure the detection and classification tasks, and this is due mainly to the rising varieties and volume of IoT malware data [18,35]. Indeed, the translation of raw data into feature vectors for use by new or conventional CNN architectures necessitates a high level of engineering and technological expertise. Furthermore, these architectures may take longer to extract features from images. In fact, to achieve excellent performance results, the detection/classification model should be trained across a large number of epochs, which takes a much longer time and several loops to obtain the optimum hyperparameters’ weights.
- Numerous related works used a single CNN architecture to achieve detectio and classification tasks [29,31], disregarding the benefits of combining several DL algorithms, which may significantly increase performance outcomes compared to those produced by a single algorithm.
- Several related works used different and sometimes multiple data augmentation techniques to improve the generalizability of their overfitted data model [27,32]. These techniques offer various benefits in terms of performance enhancement; nevertheless, they also bring several challenges that increase the complexity and the necessary resources. Some of these challenges are listed here: (1) some techniques are quite complex to apply and need reengineering efforts to fit them to the used data characteristics, (2) another challenge is determining the best data augmentation strategy, and (3) augmented data may include the same biases presented by the real dataset.

In comparison to the aforementioned malware detection and classification systems and methodologies and in order to address the weaknesses identified in these studies, our suggested approach has the following major advantages:

- Pre-trained and well-known CNN models were employed for vision-based malware classification to detect and recognize IoT-malware types, which do not necessitate pretreatment of malware’s structural properties such as binary disassembly or information extraction. The TL approach has this representational learning capacity, allowing for faster learning process based on the gained valuable knowledge.
- Because malware programmers just tamper with a small piece of the viral code to generate a new mutant, the proposed approach relied on visualizing malware as a colored image, which had the benefit of discriminating separate components/patterns of the malware binary.
- The fine-tuning of the CNN layers and hyperparameters values aided in recognizing distinct malware families and improving the classification performance of pre-trained models without the need for data augmentation methods.
- In order to improve the classification task, a variety of ensemble learning techniques was used to combine outcomes from different CNN classifiers to correctly learn the features of each malware class.
- To appropriately analyze the investigated fine-tuned CNN models, a comprehensive performance analysis is carried out by considering the seven significant assessment measures: precision, recall, specificity, F1-score, Matthews Correlation Coefficient (MCC), accuracy, and the average processing time per malware classification.
- A thorough comparison with other similar studies that used the same dataset is performed to evaluate their suggested IoT-malware detection and classification approaches and results compared to ours.

## 3. Proposed Approach

**Figure 1:**
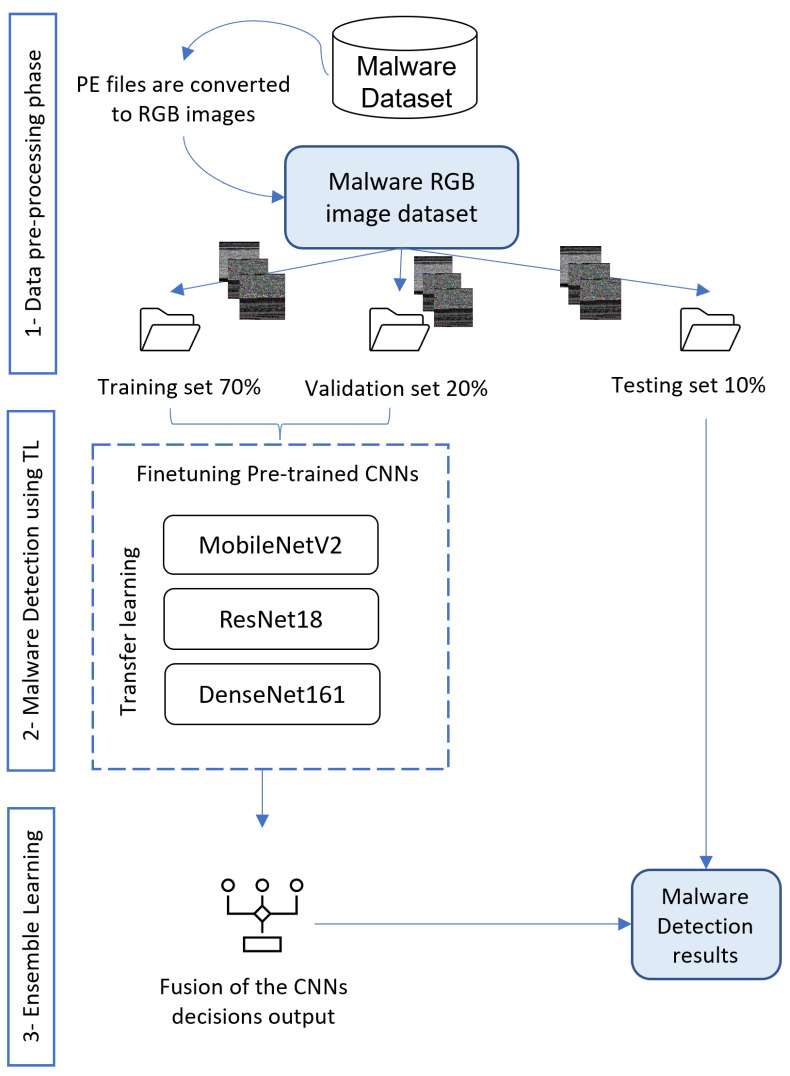
Proposed approach for malware detection and multi-classification.

This work aims to create a vision-based malware multi-classification strategy to solve the shortcomings of existing malware detection systems and provide more accurate detection and classification. The suggested approach makes use of the advantages of the deep TL methodology. It integrates the fine-tuning method as well as other ensemble techniques to improve the detection and classification performance without the need to create training models from scratch. The proposed method is divided into three main phases: data preprocessing, malware detection using TL, and fusion using ensemble learning strategies. In the preprocessing phase, the dataset is transformed from Portable Execution (PE) files to RGB images and divided into three sets for training, validation, and testing. In the second phase, three distinct pre-trained CNN architectures are loaded with their pre-learned weights, fine-tuned, and trained using the malware dataset. In our approach, we employ the models MobileNetV2, ResNet18, and DenseNet161 to apply TL. The outputs of the three models are combined in the third phase. Different ensemble methods are used to get more accurate classification results, including hard voting, soft voting, and Random Forest-based voting. The architecture of the suggested approach is depicted in Figure 1. More details about the proposed approach phases are presented in the following subsections.

### 3.1. Data Pre-Processing Phase

Employing CNNs in different applications has successfully brought intelligence to various IoT services. In this phase, the PE files obtained from malfunctioning software collected from 2017 to 2018 are converted into RGB images using the bin2png algorithm [36] to be used as input for the pre-trained CNN models.

Every three bytes of the binary file are converted into a single pixel to represent the output image. The first byte is encrypted and presented in a red channel, the second byte in a green channel, and the third byte in a blue channel. This conversion process is summarized in Algorithm 1.
**Algorithm 1** Conversion of PE files to RGB images1:**Input:** Binary file, Image dimensions.2:**Output:** RGB images.3:**Begin**4:img = new RGB Image with the specified dimensions5:pixels = load img, row = 0, column = −16:**while** read binary file is True: **do**7:    bytes = read 3 bytes from the binary file8:    column += 1, row += 19:    color = [bytes [0], bytes [1], bytes [2]]10:  pixels [column, row] = tuple (color)11:**end while**12:save img in PNG format13:**Return** RGB images

### 3.2. Malware Detection Using Transfer Learning

Using the TL approach, this phase attempts to leverage previously acquired information to handle the challenge of detecting and classifying various types of malware. When compared to models developed and trained from scratch, the adoption of pre-trained CNN architectures would speed up and improve the learning process, take less time, and use less computer resources and data [13]. We employed three distinct pre-trained CNN models in this study: ResNet18, MobileNetV2, and DenseNet161. The architecture of each of these models is detailed in the subsections that follow.

#### 3.2.1. ResNet18

ResNet18 is a CNN model proposed by He et al. in [37] and consists of 18 layers. A pre-trained ResNet18 version is available for TL, which is trained over the ImageNet dataset and able to categorize 1000 different classes. This model includes a residual learning framework that enables a more accessible network training. As a consequence, ResNet18 is widely used for TL. It provides a more straightforward training process than other CNN architectures; thus it is able to achieve good performance.

#### 3.2.2. MobileNetV2

MobileNetV2 [38] is an updated version of the MobileNet CNN architecture. This new architecture is built on an inverted residual structure in bottleneck layers, including convolutional blocks. A skip connection technique links each convolutional block’s beginning and ending points. The MobileNetV2 could access older activations that have not been updated in each convolutional block using the skip connection approach. Besides, MobileNetV2 outperforms most of the previous models in terms of performance, and it is also computationally affordable.

#### 3.2.3. DenseNet161

DenseNet161 [39] is a very deep CNN model in which the connection between layers is with a feed-forward design. Each layer in this model catches its input from the preceding layers’ feature maps, and its output feature maps are utilized as input for the subsequent layers. This procedure came up with a considerable reduction in parameters number, the performance efficiency with the reused feature maps, and the alleviating of vanishing-gradient problems. However, this model has a considerable number of layers which makes the time of the training process much longer compared to the other architectures.

### 3.3. Fusion Using Ensemble Learning Strategies

Ensemble learning is a powerful approach for merging the outputs of DL models to improve the accuracy [15,16,40]. This is often associated with the established concept that incorporating several DL models leads to higher outcomes as compared to the performance of a single DL model. Following this concept, we employ the hard voting, soft voting, and random forests-based classifier to combine the three CNNs models’ outputs.

#### 3.3.1. Hard Voting

Hard voting is defined as the majority voting that outputs the class with the most n votes [40]. This method’s core concept is to choose the final output class based on the most commonly anticipated one. Each model makes a classification prediction, and the results are recorded in a vector: [$R_{1}\left(x\right)$, $R_{2}\left(x\right)$, … , $R_{n}\left(x\right)$], where n is the number of classifiers. The voting concept is then applied to determine the output class y of a test image based on the most often predicted class in the vector by applying Equation (1).

(1) $$Y=mode\left[R_{1}\left(x\right),R_{2}\left(x\right),\ldots ,R_{n}\left(x\right)\right]$$

#### 3.3.2. Soft Voting

Soft voting classifies test data based on the projected probability *P* produced by all classifiers [40]. The average probability is calculated for each class.

Let us suppose we have the following models: $M=m_{1},m_{2},\ldots ,m_{j}$ utilized for multi-classification, the average probability of each class is obtained using Equation (2).

(2) $$P_{mean}\left(i_{j}|x\right)=\frac{1}{n}\sum_{z=1}^{n}Pm_{z}\left(i_{j}|x\right)$$

After that, the output class of the tested *x* is then determined using Equation (3), taking into consideration the greatest probability.

(3) $$Y=argmax[P_{mean}\left(i_{0}|x\right),\ldots ,P_{mean}\left(i_{j}|x)\right]$$

#### 3.3.3. Stacking Strategy

Another ensemble technique proposed to integrate the learning patterns of several models using a high-level meta learner to get more appropriate classification accuracy is the stacking strategy [41]. The primary idea underlying stacking is to combine knowledge from a collection of learned models. This study presents a novel neural network that uses the models’ prediction outputs as input, contains a hidden dense layer, and concludes with an output layer. This neural network with a multi-head input layer embeds single learned models. This network is then trained to learn how to optimally combine the sub-model predictions, resulting in a single stacking ensemble model. The models are initially loaded as a list to be used as inputs to the stacking ensemble model. Furthermore, the layers of these models are frozen so that they cannot be learned, and their weights remain fixed during the stacking ensemble model’s training. We’ll next create a hidden layer to interpret this “input” to the meta-learner, as well as an output layer to generate its own probabilistic prediction. Stacking ensemble models often outperform single trained models stacked in the first layer and decrease generalization error.

#### 3.3.4. Random Forests-Based Voting

We used the Random Forests (RF) classifier [42] to combine the three DL CNNs $\left(M_{j}\right)$. Let’s have *x* as a given input network data that consists of n columns $x=x_{1},x_{2},\ldots ,x_{n}$, each model $m_{j}$ forecasts the probability values $P=p_{1},p_{2},\ldots ,p_{n}$ of each class *y*. Using the RF classifier, the $M_{j}$ probability values are combined to generate an ensemble prediction function f(x), which uses predictions as voting on each model’s labels, as it is illustrated in Equation (5). Each probability is considered as a vote from each model. The label with the high confidence is provided as an output.

(4) $$f\left(x\right)=argmax_{y\in Y}\sum_{j=1}^{J}I\left(y=p_{i}\left(x\right)\right)$$

## 4. Experiments

This section provides and analyses the outcomes of TL and ensemble learning strategies applied to a visual-based IoT-malware dataset.

### 4.1. Experimental Setup

The implementation of the proposed algorithm is investigated using a machine with the following specifications: an Intel(R) Core(TM) i7-8565U CPU @ 1.80 GHz 1.99 GHz processor, and a 16 GB RAM running Windows 11 with an NVIDIA GeForce MX graphics card. The Jupyter Notebook [43] provided by Anaconda distribution [44] was utilized for encoding all of the tested DL models using Python 3.8 [45]. The PyTorch library [46], which is an open-source, extensible, and modular DL framework was employed to implement the TL and fine-tune the CNN models.

The primary purpose of the suggested approach is to identify and characterize the type of malware appropriately. Three distinct CNN architectures based on TL were used for this goal. The CNNs were trained over a period of 35 epochs. We used the Adam optimizer [47] with a learning rate of 1e-3 and the cross-entropy loss function to configure the models. The size of the input images was (224 × 224) pixels, while the batch size was 64. Table 1 shows the hyperparameters used for models training.

To guarantee that the proposed approach yields the best results, extensive experiments on the MaleVis dataset with a wide variety of hyperparameters are carried out to determine the appropriate hyperparameters. Using the hit and try strategy, we identified precise values of these hyperparameters. This strategy is rigorous, and it has been widely used in a variety of recent studies offering ML and DL-based solutions [48,49,50], since optimization methods and techniques incur additional computing costs.

### 4.2. Dataset

We utilized the “Malware Evaluation with Vision” MaleVis dataset for the experiments [51,52]. This dataset contains 14,226 RGB converted images resized in 2 different square-sized resolutions (i.e., 224 × 224 and 300 × 300 pixels) representing 26 distinct families. It includes 25 classes of five common malware types, namely Adware, Trojan, Virus, Worm, Backdoor, and one benign class. In the following, we provide a brief definition of each of these malware types [53]:

- Adware is unwanted program that displays advertising on your screen, usually through a web browser.
- A Trojan, is malicious code is designed to harm, disrupt, steal, or in general harm your data or network.
- A virus is a sort of malware that repeats itself by embedding its code into other programs.
- A worm is a standalone malicious computer software that spreads by using a computer network to propagate to other systems.
- A backdoor is a sort of malware that bypasses standard authentication mechanisms to gain access to a system.

**Figure 2:**
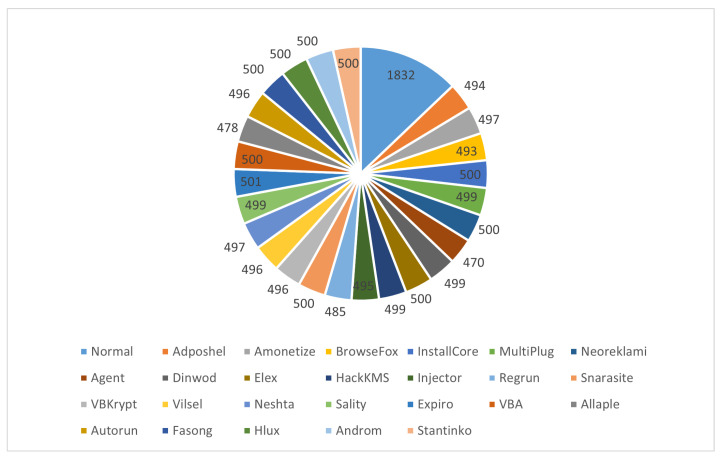
The distribution of samples for each class in the Malevis dataset.

**Figure 3:**
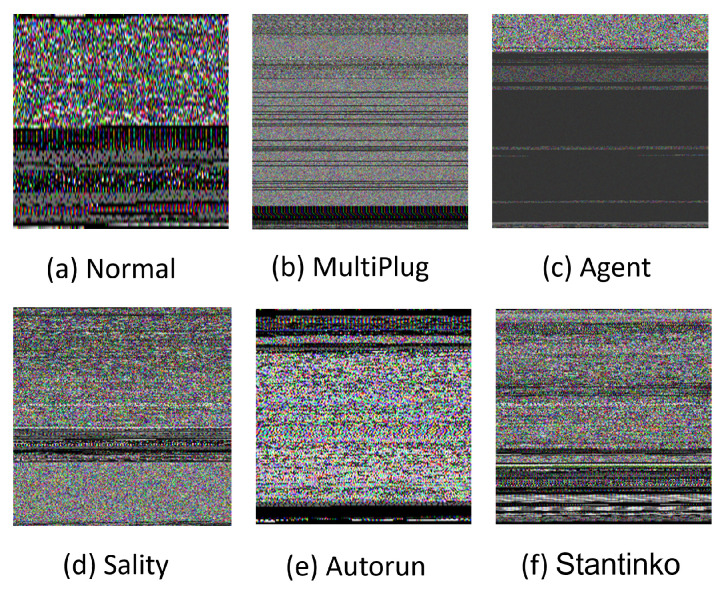
Malware RGB images from different malware families and the benign class provided by the Malevis dataset.

Table 2 depicts the distribution of the used dataset. As seen in Figure 2, the dataset has balanced classes for the malware types with 500 images or a close number. The Normal class, on the other hand, has more samples, with 1832 images. For experiments, the dataset was divided into 70 %, 20%, and 10% for training, validation, and testing, respectively. Figure 3 presents examples of RGB malware images of the MaleVis dataset for Normal, MultiPlug, Agent, Sality, Autorun, and Stantinko cases.

### 4.3. Performance Metrics

In our study, the model’s efficiency for malware detection and classification tasks is measured using the accuracy metric. This metric shows the proportion of correct predictions out of all predictions made. Accuracy is the most intuitive measure used for DL models’ assessment; that is why it is commonly employed. However, it is often important and advantageous to dig deeper when evaluating DL models. Accuracy is a meaningful metric to use when dealing with a balanced dataset, but it may produce good results for datasets with unbalanced classes despite the model’s poor performance. Consequently, employing other metrics such as precision, recall, and F1-score gives additional information about the model’s performance by taking into account the type of errors made by the model rather than just the number of prediction errors made. These metrics are widely used in the field of malware detection and classification that leverages DL models [26,28,34].

We calculate the precision, recall, specificity, F1-score, MCC, accuracy, loss, and average processing time per malware classification metrics to assess the performance of the developed classifiers. Each statistical metric is presented with its corresponding mathematical representation in Equations (5)–(10), where:

- True Positive (*TP*): Malware (positive) is the expected case, and the prediction is correct;
- True Negative (*TN*): Normal (negative) is the expected case, and the prediction is correct;
- False Positive (*FP*): Malware (positive) is the expected case, and the prediction is incorrect;
- False Negative (*FN*): Normal (negative) is the expected case, and the prediction is incorrect;

**Precision (Pre):** it is employed to measure the model accuracy in categorizing a sample as positive.

(5) $$Precision=\frac{TP}{TP+FP}$$

**Recall:** it is employed to assess the model ability to identify the positive samples.

(6) $$Recall=\frac{TP}{TP+FN}$$

**Specificity (Spec):** it is used to assess the model capacity to identify negative samples.

(7) $$Specificity=\frac{TN}{TP+FN}$$

**F1-score:** it leverages both the precision and recall metrics to get a value-added metric used for performance verification.

(8) $$F1-score=\frac{2*Precision*Recall}{Precision+Recall}$$

**MCC:** it is obtained by calculating the correlation coefficient across the observed and predicted classifications.

(9) $$MCC=\frac{\left(TP*TN)-(FP*FN\right)}{\sqrt{\left(TP+FP)(TP+FN)(TN+FP)(TN+FN\right)}}$$

**Accuracy (Acc):** it is employed to generally assess the model performance across all classes.

(10) $$Accuracy=\frac{TP+TN}{TP+TN+FP+FN}$$

**Loss:** is used to compute the error value and assess how effectively the model handles the data.

**Average Processing Time (APT) per malware classification:** denotes the average processing time per malware to obtain the final classification result.

### 4.4. Results and Discussion

This subsection summarizes the findings of the experiments performed on the MaleVis dataset and provides a comparative analysis with recent published works that have used in their experiments the same dataset.

#### 4.4.1. Experimental Results

The primary goal of the proposed approach is to detect and classify the malware cases correctly. In this respect, three different pre-trained CNN architectures have been employed.

During training, a 5-fold cross-validation is performed to improve models’ performance. To validate the model generalization measures, the k-fold cross-validation technique [54] is used. The fundamental concept behind this technique is to divide the data into k groups of similar size. The model is then fitted using a single data group for validation, and the remaining data groups are kept for training. This cycle is repeated k times, with a new distribution of data groups chosen for each iteration. Finally, the final model’s results are obtained by computing the average of all metrics acquired across the k iterations. Table 3 illustrates the accuracy achieved when training the three CNNs models using a 5-fold cross-validation. The MobileNetV2 model produced the best results, with an accuracy of 97.67%, whereas ResNet 18 provided the worst results. DenseNet161 had a 96.66% accuracy rate. The validation accuracy for all models is above 95%, according to the results in Table 3.

Table 4 shows the performance results of the CNN models after they have been fine-tuned and trained on the MaleVis dataset. The MobileNetV2 model clearly outperformed the others, with an accuracy of 97.67%, a precision of 97.8%, and a recall of 97.74%, followed by the DenseNet161 then the ResNet18. In general, the performance of these models is good with an accuracy that exceeded 95%.

**Figure 4:**
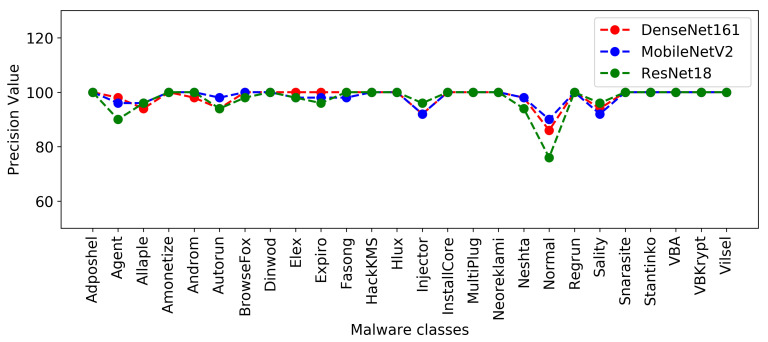
Precision of each malware class resulted from DenseNet161, MobileNetV2, and ResNet18.

**Figure 5:**
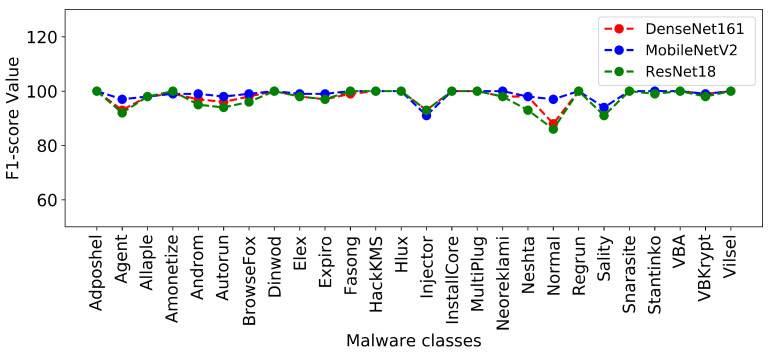
F1-score of each malware class resulted from DenseNet161, MobileNetV2, and ResNet18.

Figure 4 and Figure 5 show the plots of precision and F1-score metrics for each malware class produced by the DenseNet161, MobileNetV2, and ResNet18 models.

The suggested approach’s next step is to leverage various ensemble learning strategies to fuse the models’ outputs, significantly increasing malware detection and classification. Four distinct strategies have been utilized in this context: hard voting, soft voting, staking strategy, and random forests-based voting. Table 5 shows the performance results of the obtained classifiers. The combined classifiers clearly outperformed the individual models in terms of accuracy. Furthermore, the time required to obtain a prediction using the hard, soft, and random forest strategies is quite close, and these strategies are faster than the stacking ensemble strategy.

We conclude that the random forest-based voting classifier outperforms the hard and soft voting strategies and the stacking ensemble strategy in terms of detection and classification performance. It achieves a precision of 98.74%, a recall of 98.67%, a specificity of 98.79%, an F1-score of 98.70%, a MCC of 98.65%, an accuracy of 98.68%, and an average processing time per malware classification of 672 ms. As a result, we chose the random forest-based voting classier as the recommended ensembling strategy.

**Figure 6:**
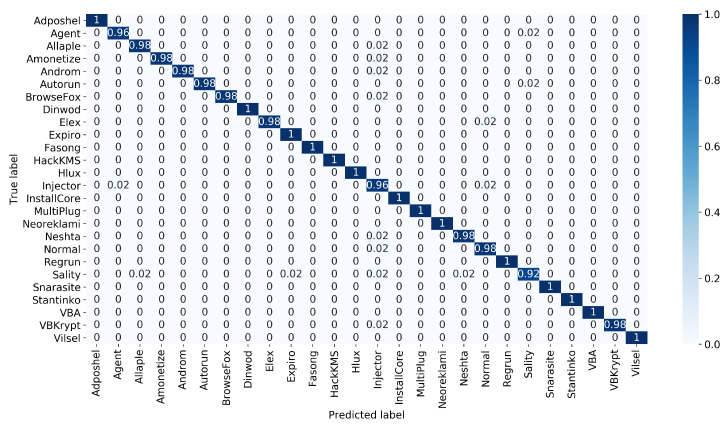
The normalized confusion matrix of the proposed approach.

**Figure 7:**
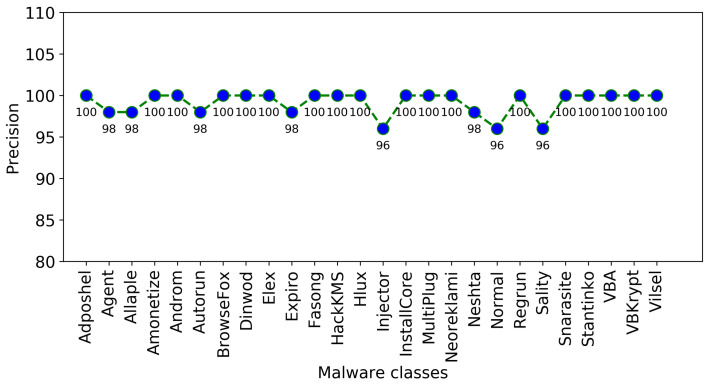
Precision of each malware class using the proposed approach.

**Figure 8:**
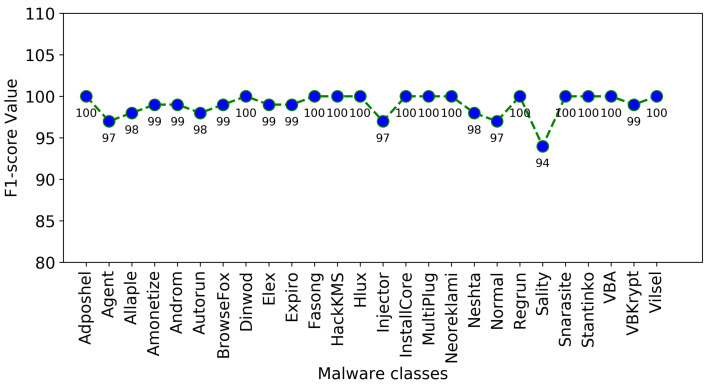
F1-score of each malware class using the proposed approach.

The confusion matrix of the proposed approach is presented in Figure 6, Figure 7 and Figure 8 illustrate the plots of precision and F1-score of each malware class obtained using the proposed approach, where it is obvious that the random forest-based voting classifier significantly improves the outcomes of these performance measures.

In conclusion, the various ensemble learning strategies used give good performance that surpasses the performance of sub-models. As a result, we conclude that combining several DL models produces more valuable results than using a single DL model.

**Figure 9:**
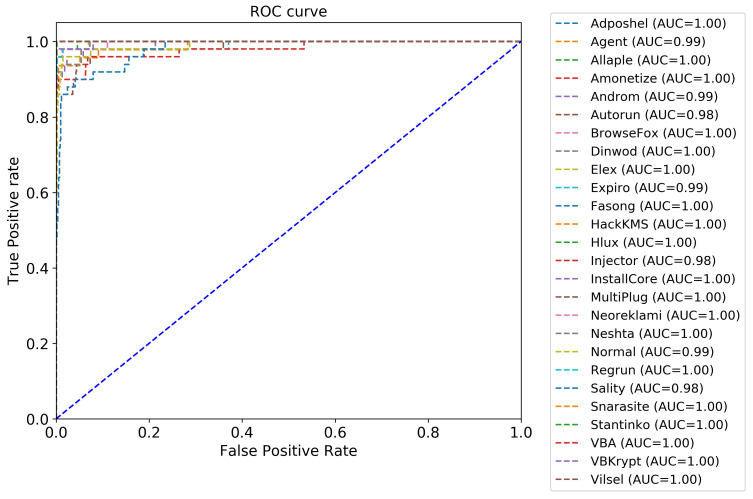
Plots of TP rate versus FP rate of the proposed approach.

**Figure 10:**
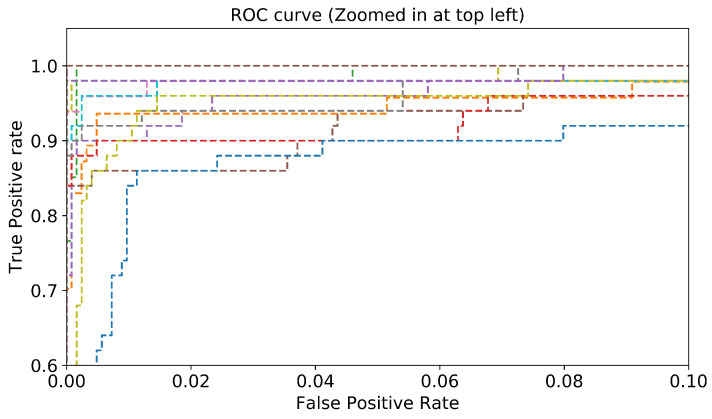
Zoomed-in version of Figure 9.

Another essential evaluation metric for validating the performance of the proposed classifier is the Receiver Operating Characteristic (ROC) curve. The ROC curve is developed by plotting the True Positive Rate (TPR), which is the recall, on the y-axis versus the False Positive Rate (FPR), which is the specificity, on the x-axis. Figure 9 depicts the ROC curve of the proposed approach, where 26 (number of classes) ROC curves are presented. Figure 10 is a zoomed-in version of Figure 9. Most of the malware classes (19 classes) obtained an AUC of 100%. A large area under the curve is observed for the other classes, where the AUC value ranges between 98% and 99%. Consequently, we conclude that the proposed approach performs well in detecting and classifying the different malware classes.

#### 4.4.2. Comparison with Similar Works

MaleVis is a new dataset that was released in 2019. With this dataset, many ML, DL, and TL algorithms have been presented to develop smart classifiers for effective malware detection. In this study, we used both TL and ensemble learning to create a multi-classification approach that effectively detected and classified the 25 malware types in the MaleVis dataset. The proposed approach was compared with current methods in the literature.

Table 6 summarizes the comparison of performance results in terms of precision, recall, F1-score, accuracy, and average processing time per malware classification. According to Table 6, our proposed approach outperforms the state-of-the-art approaches, with the best recall (98.67%), F1-score (98.70%), and accuracy (98.68%). Although [35] had the best precision rate, our approach outperformed it in terms of recall, F1-score, and accuracy. These significant improvements are attributed to the usage of TL and ensembling strategies, which aid in producing excellent outcomes, with 0.47% accuracy improvement, 0.55% F1-score improvement, and 0.93% recall improvement by reusing and fusing the knowledge collected from previously trained models. Furthermore, the notion of merging several CNNs assists in achieving optimal outcomes that outperform single models.

The suggested classifier has a response time of less than one second (672 ms) and a 98.7% accuracy in predicting the outcome of a malware attack. Work [18] produced an output after 5090 ms. In [35], it was stated that the suggested classifier required a long computation time to produce an output; however, in the other comparison research, the processing time was not mentioned. The results demonstrate that the proposed approach takes less time to obtain an output from the attack samples when compared to other malware attack detection systems.

#### 4.4.3. Discussion

This paper aims to investigate the use of TL to detect and classify IoT-Malware. Three TL techniques have been used, namely, ResNet18, MobileNetV2, and DenseNet161. In addition, several ensemble learning approaches have been employed (i.e., hard voting, soft voting, stacking strategy, and random forests) to combine the results of the three CNNs models’ outputs. A public dataset, MaleVis, made of 14,226 RGB images representing 25 malware classes and one benign class, is considered to validate the proposed approach. The obtained results show that the proposed approach has improved malware detection.

As shown in Table 5, the random forest-based voting achieved 98.74%, 98.67%, 98.79%, 98.70%, and 98.65%, 98.68%, 672 ms, for the seven performance metrics: precision, recall, specificity, F1-score, MCC, accuracy, and average processing time per malware classification, respectively. Random forest-based voting outperformed hard voting, soft voting, and the stacking ensemble model. Extensive experiments have been conducted to evaluate the performance of the proposed method in analyzing the different classes of malware types. In addition, the proposed approach was compared with five current related works in the literature that used the same Malevis dataset. Results showed that the proposed approach achieved the best results for recall, F1-score, and accuracy metrics compared to related works.

The effectiveness of ML and DL algorithms in modeling and evaluating spatial and temporal fluctuations of data environments has been proved in different domains [57,58]. In fact, feature representation learning enables these algorithms to properly capture spatial and temporal correlations. These findings are also transposed to the malware detection and classification field. Due to the rapid learning of distinct features through malware classes, the model can distinguish between them and accurately recognize different types of attacks. Consequently, the proposed approach is able to take into account the environment’s spatial and temporal variety and can identify and categorize efficiently distinct learned attack types even when the time or the surroundings change.

This study highlighted the effectiveness of combining several pre-trained CNN models using different ensemble strategies, such as voting, stacking, and random forest, to improve the detection and classification of malware. This choice is justified by the benefits afforded by the ensembling strategy [15,16]. In fact, when compared to a single contributing model, an ensemble can produce greater predictions and yield better performance results. Furthermore, an ensemble is able to reduce the spread or dispersion of the prediction errors generated by the contributing models. The experimental results that are obtained in the present study illustrate that combining the features of several TL models predicts better malware classes than applying a single TL method on the same dataset. A performance comparison was carried out in order to select the most effective ensemble learning strategy. In this comparison, random forest voting surpassed other strategies such as hard and soft voting as well as the stacking approach. Indeed, there are several issues related to hard and soft voting, mainly disregarding the correct decision of the minority, and selecting the number of candidate classifiers. For the stacking strategy, training may be time-intensive, and it does not bring performance improvements in the case of small-size training datasets. The previous arguments make the random forests-based approach the suitable solution that overcomes the limitations of the voting and stacking strategies. Moreover, random forests classifier presents numerous advantages, mainly [59]: (1) it helps to increase accuracy by reducing overfitting in decision trees, (2) it is capable of dealing with both classification and regression tasks, (3) it is able to manage both categorical and continuous data, (4) it substitutes missing values in the learned data automatically, and (5) it does not necessitate data normalization because it employs a rule-based approach. All of these benefits make the random forests-based approach an excellent choice for complex tasks like IoT-malware detection and multi-classification. This explains and consolidates the experimental findings of this study.

## 5. Conclusions and Future Research Directions

The IoT is a cutting-edge networking concept that connects a wide range of devices. To operate correctly, each of those devices needs many pieces of software or programs to be installed. Even though these programs have unrivaled analytical and decision-making capabilities, their vulnerabilities can be exploited, resulting in a wide range of security threats and repercussions. Consequently, understanding IoT-based applications is critical for addressing security flaws through detection and classification. Recently, a promising direction has been explored that depends on artificial intelligence algorithms, notably DL models, which have demonstrated their efficacy by offering remarkable performance in detecting malware. Therefore, this study introduced a novel detection and multi-classification approach for IoT-malware that uses the advantages of deep TL methodology and integrates the fine-tuning method and a set of ensembling strategies to improve detection and classification performance without having to create training models from scratch. ResNet18, MobileNetV2, and DenseNet161 were the three deep CNN models employed. To merge the outputs of these models, multiple ensemble learning algorithms, namely hard voting, soft voting, and random forests, were used. MaleVis, a publicly available dataset, is used in the experiments to test and validate the suggested approach. MaleVis is a collection of more than 14,000 RGB-converted images that represent 25 malware classes and one benign class. The obtained results reveal that our proposed approach exceeds current state-of-the-art methods in terms of detection and classification performance; it achieves a precision of 98.74%, recall of 98.67%, f1-score of 98.70%, MCC of 98.65%, an accuracy of 98.68%, and an average processing time per malware classification of 672 ms.

Promising extensions of the present study are divided into two major parts: practical and theoretical. For the practical part, we intend to: (1) test our model by using other balanced and unbalanced datasets that include spatial and temporal variations since our approach was only tested on a single dataset in the current study, and (2) incorporate additional types of pre-trained CNN models to improve performance outcomes. For the theoretical extensions, we are planning to: (1) propose a new DL architecture based on the generative adversarial networks and transformers to improve IoT-malware detection and multi-classification performance by expanding unbalanced malware datasets, (2) adopt the federated learning approach, which, thanks to its low complexity and distributed nature, ensures the deployment of ML and DL classifiers on IoT sensors instead of manipulating IoT data at a centralized server; this approach will offer real-time IoT-malware detection and classification solutions enhancing cyberattack mitigation and prevention, and finally, (3) extend the proposed approach to ensure the detection and the multi-classification of malicious software in the Industrial Internet of Things (IIoT) environments.

## Figures and Tables

**Table 1:** The used hyperparameters for the models’ configuration.

Models’ Training Hyperparameters	Values
Batch size	64
Epochs	35
Images size	224 × 224
Learning rate	1e-3
Optimizer	Adam
Loss function	Cross-entropy

**Table 2:** The distribution of the MaleVis dataset.

Category	Type	Class	Samples	Total
Benign	-	Normal	1832	1832
Malware	Adware	Adposhel	494	2983
		Amonetize	497	
		BrowseFox	493	
		InstallCore	500	
		MultiPlug	499	
		Neoreklami	500	
	Trojan	Agent	470	4440
		Dinwod	499	
		Elex	500	
		HackKMS	499	
		Injector	495	
		Regrun	485	
		Snarasite	500	
		VBKrypt	496	
		Vilsel	496	
	Virus	Neshta	497	1997
		Sality	499	
		Expiro	501	
		VBA	500	
	Worm	Allaple	478	1974
		Autorun	496	
		Fasong	500	
		Hlux	500	
	Backdoor	Androm	500	1000
		Stantinko	500	

**Table 3:** Accuracy results of the deployed TL-based CNN models through a 5-fold cross-validation.

	Model 1: ResNet 18 Acc (%)	Model 2: MobileNetV2 Acc (%)	Model 3: DenseNet161 Acc (%)
**Iteration 1**	95.04	97.60	96.71
**Iteration 2**	95.05	97.65	96.61
**Iteration 3**	95.01	97.74	96.86
**Iteration 4**	95.03	97.70	96.65
**Iteration 5**	95.02	97.66	96.47
**Final model**	**95.03**	**97.67**	**96.66**

**Table 4:** Performance results of the deployed TL-based models.

Model	Pre (%)	Recall (%)	Spec (%)	F1-Score (%)	MCC (%)	Acc (%)	Loss	APT per Malware Classification (ms)
ResNet18	95.68	95.12	95.14	95.39	95.26	95.03	0.181	146
MobileNetV2	97.8	97.74	97.69	97.76	97.54	97.67	0.086	97
DenseNet161	96.64	96.71	96.97	96.67	96.64	96.66	0.156	480

**Table 5:** Performance results of the considered ensemble learning strategies.

Ensemble Learning	Pre (%)	Recall (%)	Spec (%)	F1-score (%)	MCC (%)	Acc (%)	APT per Malware Classification (ms)
Hard voting	97.90	97.75	97.87	97.82	97.71	97.75	712
Soft voting	98.11	97.91	97.97	98.00	97.88	97.90	681
Stacking ensemble model	98.41	98.34	98.47	98.44	98.23	98.34	1845
**Random forest-based voting** ** (Proposed approach)**	**98.74**	**98.67**	**98.79**	**98.70**	**98.65**	**98.68**	**672**

**Table 6:** Performance comparison between the proposed detection and multi-classification approach for IoT-malware using random forest voting of fine-tuning CNNs and related approaches tested on the MaleVis dataset.

Ref	Method	Precision (%)	Recall (%)	F1-score (%)	Accuracy (%)	APT (ms)
[52]	TL (DenseNet201)	not stated	not stated	not stated	97.48	not stated
[55]	Deep random forest approach	97.43	97.32	97.42	97.43	not stated
[56]	Hybrid CNN approach using AlexNet and ResNet152	97.1	94.9	94.5	96.6	not stated
[35]	TL (ShuffleNet/ DenseNet201)	**99.80**	95.61	95.37	95.01	high APT (in-loop fitness evaluation)
[18]	Pretrained DenseNet model with a reweighted categorical cross-entropy loss criterion	98.56	97.74	98.15	98.21	5090
**Proposed approach**	**Random forest-based voting classifier**	98.744	**98.67**	**98.70**	**98.98**	**672**

## Data Availability

Dataset link: https://web.cs.hacettepe.edu.tr/selman/malevis/ (accessed on 3 May 2022).

## References

[1] Ben Atitallah S., Driss M., Boulila W., Ben Ghézala H. (2020). Leveraging Deep Learning and IoT big data analytics to support the smart cities development: Review and future directions. Comput. Sci. Rev..

[2] Latif S., Driss M., Boulila W., Huma Z.E., Jamal S.S., Idrees Z., Ahmad J. (2021). Deep Learning for the Industrial Internet of Things (IIoT): A Comprehensive Survey of Techniques, Implementation Frameworks, Potential Applications, and Future Directions. Sensors.

[3] IoT Cyberattacks Escalate in 2021, According to Kaspersky. https://www.iotworldtoday.com/2021/09/17/iot-cyberattacks-escalate-in-2021-according-to-kaspersky/.

[4] Ngo Q.D., Nguyen H.T., Le V.H., Nguyen D.H. (2020). A survey of IoT malware and detection methods based on static features. ICT Express.

[5] Vignau B., Khoury R., Hallé S., Hamou-Lhadj A. (2021). The evolution of IoT Malwares, from 2008 to 2019: Survey, taxonomy, process simulator and perspectives. J. Syst. Archit..

[6] Baig M., Zavarsky P., Ruhl R., Lindskog D. (2012). The study of evasion of packed pe from static detection. Proceedings of the World Congress on Internet Security (WorldCIS-2012).

[7] Fernando D.W., Komninos N., Chen T. (2020). A study on the evolution of ransomware detection using machine learning and deep learning techniques. IoT.

[8] Bello I., Chiroma H., Abdullahi U.A., Gital A.Y., Jauro F., Khan A., Okesola J.O., Abdulhamid S.M. (2021). Detecting ransomware attacks using intelligent algorithms: Recent development and next direction from deep learning and big data perspectives. J. Ambient. Intell. Humaniz. Comput..

[9] Zakaria W.Z.A., Abdollah M.F., Mohd O., Ariffin A.F.M. The rise of ransomware. Proceedings of the 2017 International Conference on Software and e-Business.

[10] Loey M., Manogaran G., Taha M.H.N., Khalifa N.E.M. (2021). A hybrid deep transfer learning model with machine learning methods for face mask detection in the era of the COVID-19 pandemic. Measurement.

[11] Ben Atitallah S., Driss M., Boulila W., Ben Ghezala H. (2022). Randomly initialized convolutional neural network for the recognition of COVID-19 using X-ray images. Int. J. Imaging Syst. Technol..

[12] Ben Atitallah S., Driss M., Boulila W., Koubaa A., Ben Ghezala H. (2022). Fusion of convolutional neural networks based on Dempster–Shafer theory for automatic pneumonia detection from chest X-ray images. Int. J. Imaging Syst. Technol..

[13] Tan C., Sun F., Kong T., Zhang W., Yang C., Liu C. (2018). A survey on deep transfer learning. Proceedings of the International Conference on Artificial Neural Networks.

[14] Vrbančič G., Podgorelec V. (2020). Transfer learning with adaptive fine-tuning. IEEE Access.

[15] Krawczyk B., Minku L.L., Gama J., Stefanowski J., Woźniak M. (2017). Ensemble learning for data stream analysis: A survey. Inf. Fusion.

[16] Sagi O., Rokach L. (2018). Ensemble learning: A survey. Wiley Interdiscip. Rev. Data Min. Knowl. Discov..

[17] Nisa M., Shah J.H., Kanwal S., Raza M., Khan M.A., Damaševičius R., Blažauskas T. (2020). Hybrid malware classification method using segmentation-based fractal texture analysis and deep convolution neural network features. Appl. Sci..

[18] Hemalatha J., Roseline S.A., Geetha S., Kadry S., Damaševičius R. (2021). An efficient densenet-based deep learning model for malware detection. Entropy.

[19] Yan P., Yan Z. (2018). A survey on dynamic mobile malware detection. Softw. Qual. J..

[20] Souri A., Hosseini R. (2018). A state-of-the-art survey of malware detection approaches using data mining techniques. Hum. Centric Comput. Inf. Sci..

[21] Sharma S., Khanna K., Ahlawat P. (2022). Survey for Detection and Analysis of Android Malware (s) Through Artificial Intelligence Techniques. Cyber Security and Digital Forensics.

[22] Lo W.W., Yang X., Wang Y. (2019). An xception convolutional neural network for malware classification with transfer learning. Proceedings of the 2019 10th IFIP International Conference on New Technologies, Mobility and Security (NTMS).

[23] Davuluru V.S.P., Narayanan B.N., Balster E.J. (2019). Convolutional neural networks as classification tools and feature extractors for distinguishing malware programs. Proceedings of the 2019 IEEE National Aerospace and Electronics Conference (NAECON).

[24] Zhao Y., Cui W., Geng S., Bo B., Feng Y., Zhang W. (2020). A malware detection method of code texture visualization based on an improved faster RCNN combining transfer learning. IEEE Access.

[25] Narayanan B.N., Davuluru V.S.P. (2020). Ensemble malware classification system using deep neural networks. Electronics.

[26] Vasan D., Alazab M., Wassan S., Safaei B., Zheng Q. (2020). Image-Based malware classification using ensemble of CNN architectures (IMCEC). Comput. Secur..

[27] Olowoyo O., Owolawi P. (2020). Malware classification using deep learning technique. Proceedings of the 2020 2nd International Multidisciplinary Information Technology and Engineering Conference (IMITEC).

[28] Awan M.J., Masood O.A., Mohammed M.A., Yasin A., Zain A.M., Damaševičius R., Abdulkareem K.H. (2021). Image-Based Malware Classification Using VGG19 Network and Spatial Convolutional Attention. Electronics.

[29] Sudhakar, Kumar S. (2021). MCFT-CNN: Malware classification with fine-tune convolution neural networks using traditional and transfer learning in internet of things. Future Gener. Comput. Syst..

[30] Carletti V., Greco A., Saggese A., Vento M. Robustness evaluation of convolutional neural networks for malware classification. Proceedings of the Italian Conference on Cybersecurity (ITASEC).

[31] Bouchaib P., Bouhorma M. Transfer Learning and Smote Algorithm For Image-Based Malware Classification. Proceedings of the 4th International Conference on Networking, Information Systems & Security.

[32] Khetarpal A., Mallik A. (2021). Visual Malware Classification Using Transfer Learning. Proceedings of the 2021 Fourth International Conference on Electrical, Computer and Communication Technologies (ICECCT).

[33] El-Shafai W., Almomani I., AlKhayer A. (2021). Visualized malware multi-classification framework using fine-tuned CNN-based transfer learning models. Appl. Sci..

[34] Kumar S., Janet B. (2022). DTMIC: Deep transfer learning for malware image classification. J. Inf. Secur. Appl..

[35] Wong W., Juwono F.H., Apriono C. (2021). Vision-Based Malware Detection: A Transfer Learning Approach Using Optimal ECOC-SVM Configuration. IEEE Access.

[36] Bin To PNG Conversion. https://web.cs.hacettepe.edu.tr/~selman/malevis/bin2png.py.

[37] He K., Zhang X., Ren S., Sun J. Deep residual learning for image recognition. Proceedings of the IEEE Conference on Computer Vision and Pattern Recognition.

[38] Sandler M., Howard A., Zhu M., Zhmoginov A., Chen L.C. Mobilenetv2: Inverted residuals and linear bottlenecks. Proceedings of the IEEE Conference on Computer Vision and Pattern Recognition.

[39] Huang G., Liu Z., Van Der Maaten L., Weinberger K.Q. Densely connected convolutional networks. Proceedings of the IEEE Conference on Computer Vision and Pattern Recognition.

[40] Du K.L., Swamy M. (2019). Combining Multiple Learners: Data Fusion and Ensemble Learning. Neural Networks and Statistical Learning.

[41] Jiang M., Liu J., Zhang L., Liu C. (2020). An improved Stacking framework for stock index prediction by leveraging tree-based ensemble models and deep learning algorithms. Phys. Stat. Mech. Its Appl..

[42] Cutler A., Cutler D.R., Stevens J.R. (2012). Random forests. Ensemble Machine Learning.

[43] Jupyter: Free Software, Open Standards, and Web Services for Interactive Computing across all Programming Languages. https://jupyter.org/.

[44] Anaconda. https://www.anaconda.com/.

[45] Python Programming Language. https://www.python.org/.

[46] An Open Source Machine Learning Framework: PyTorch. https://pytorch.org/.

[47] Kingma D.P., Mohamed S., Jimenez Rezende D., Welling M. (2014). Semi-supervised learning with deep generative models. Adv. Neural Inf. Process. Syst..

[48] Rehman M.U., Shafique A., Khalid S., Driss M., Rubaiee S. (2021). Future forecasting of COVID-19: A supervised learning approach. Sensors.

[49] Huma Z.E., Latif S., Ahmad J., Idrees Z., Ibrar A., Zou Z., Alqahtani F., Baothman F. (2021). A hybrid deep random neural network for cyberattack detection in the industrial internet of things. IEEE Access.

[50] Driss M., Almomani I., Ahmad J. (2022). A federated learning framework for cyberattack detection in vehicular sensor networks. Complex Intell. Syst..

[51] MaleVis Dataset. https://web.cs.hacettepe.edu.tr/~selman/malevis/.

[52] Bozkir A.S., Cankaya A.O., Aydos M. (2019). Utilization and comparision of convolutional neural networks in malware recognition. Proceedings of the 2019 27th Signal Processing and Communications Applications Conference (SIU).

[53] Shalaginov A., Dyrkolbotn G.O., Alazab M. (2021). Review of the malware categorization in the era of changing cybethreats landscape: Common approaches, challenges and future needs. Malware Analysis Using Artificial Intelligence and Deep Learning.

[54] Refaeilzadeh P., Tang L., Liu H. (2009). Cross-validation. Encycl. Database Syst..

[55] Roseline S.A., Geetha S., Kadry S., Nam Y. (2020). Intelligent vision-based malware detection and classification using deep random forest paradigm. IEEE Access.

[56] Aslan Ö., Yilmaz A.A. (2021). A new malware classification framework based on deep learning algorithms. IEEE Access.

[57] Xu H., Ding W., Shen W., Wang J., Yang Z. (2022). Deep convolutional recurrent model for region recommendation with spatial and temporal contexts. Hoc Netw..

[58] Teng Z., Xing J., Wang Q., Zhang B., Fan J. (2019). Deep spatial and temporal network for robust visual object tracking. IEEE Trans. Image Process..

[59] Fawagreh K., Gaber M.M., Elyan E. (2014). Random forests: From early developments to recent advancements. Syst. Sci. Control Eng. Open Access J..

